# Nd:YAG fourth harmonic (266-nm) generation for corneal reshaping procedure: An ex-vivo experimental study

**DOI:** 10.1371/journal.pone.0260494

**Published:** 2021-11-29

**Authors:** Ibrahim Abdelhalim, Omnia Hamdy, Aziza Ahmed Hassan, Salah Hassab Elnaby

**Affiliations:** 1 Engineering Applications of Lasers Dept., National Institute of Laser Enhanced Sciences, Cairo University, Giza, Egypt; 2 Medical Applications of Lasers Dept., National Institute of Laser Enhanced Sciences, Cairo University, Giza, Egypt; Mohanlal Sukhadia University, INDIA

## Abstract

Corneal reshaping is a common medical procedure utilized for the correction of different vision disorders relying on the ablation effect of the UV pulsed lasers, especially excimer lasers (ArF) at 193 nm. This wavelength is preferred in such medical procedures since laser radiation at 193 nm exhibits an optimum absorption by corneal tissue. However, it is also significantly absorbed by the water content of the cornea resulting in an unpredictability in the clinical results, as well as the high service and operation cost of the commercial ArF excimer laser device. Consequently, other types of solid-state UV pulsed lasers have been introduced. The present work investigates the ablation effect of solid-state laser at 266 nm in order to be utilized in corneal reshaping procedures. Different number of pulses has been applied to Polymethyl Methacrylate (PMMA) and ex-vivo rabbit cornea to evaluate the ablation effect of the produced laser radiation. PMMA target experienced ellipse-like ablated areas with a conical shape in the depth. The results revealed an almost constant ablation area regardless the number of laser pulses, which indicates the stability of the produced laser beam, whereas the ablation depth increases only with increasing the number of laser pulses. Examination of the ex-vivo cornea showed a significant tissue undulation, minimal thermal damage, and relatively smooth ablation surfaces. Accordingly, the obtained 266-nm laser specifications provide promising alternative to the traditional 193-nm excimer laser in corneal reshaping procedure.

## Introduction

Laser technology has been widely used in many medical specialties including surgery [1], dermatology [2], and ophthalmology [3]. It is considered safe and efficient tool in different ophthalmologic procedures [4] especially corneal refractive surgery [5]. Cornea is a transparent avascular tissue that is responsible for approximately two-thirds of the total focusing power of the eye [6]. The Cornea consists of five layers; epithelium, Bowman’s layer, stroma, Descemet’s membrane, and the endothelium. Epithelium is the frontal protective layer of the cornea; it can heal itself unlike stroma. Stroma is the thickest layer of the cornea, it represents about 90% of the corneal thickness [7]. Accordingly, any change in the curvature of cornea (corneal reshaping) makes a significant effect on the total refractive power.

Corneal reshaping procedure is proposed to solve many vision problems such as myopia, hyperopia, and astigmatism [8–11]. This process is accomplished relying on the photo-ablation effect of the UV pulsed lasers [1, 12–15]. The UV laser radiation is mostly absorbed by the main chromophores in cornea and affects the polypeptide bond between the amino acids in the collagen fibers of the stroma layer [1, 16, 17]. Different UV laser sources have been proposed for corneal reshaping purposes, however, ArF excimer laser at 193 nm is commonly used as well as KrCl at 223 nm and KrF at 248 nm [18]. Excimer laser at 193 nm light provides precise corneal photo-ablation effect with less collateral damage as compared to that at longer wavelengths. Nevertheless, ArF excimer laser has some drawbacks including the high price and toxicity of its active medium (ArF) [19]. Therefore, solid-state lasers were proposed as alternative sources of such UV radiation [20].

Solid-state lasers have some theoretical and practical advantages as compared to excimer laser systems. That is, the emission wavelengths of the solid-state lasers can be closer to the absorption peak of corneal collagen and less sensitive to corneal hydration as compared to those emitted from excimer lasers [18, 21, 22]. Moreover, solid-state lasers exhibit higher pulse-to-pulse energy stability, lower maintenance costs, and smaller spot size [23]. The latter minimizes the mechanical stress of the cornea during ablation, which is associated with endothelial cell changes [24, 25].

However, only few solid-state laser platforms are commercially available [24, 26–29]. Researchers used the fifth harmonic of Nd:YAG laser (emits at 213 nm) for both in-vitro and in-vivo investigations [24, 26–29]. These investigations demonstrated smooth ablation surfaces, whereas the clinical course and the histopathologic findings were similar to those of excimer laser photorefractive keratectomy (PRK). As a result of the strong corneal absorption of the laser radiation in the far UV region (190–220 nm), high precision of corneal ablation without damage to the adjacent tissue is obtained [30, 31]. Moreover, laser radiation at 213 nm is less sensitive to corneal hydration than that at 193 nm. Thus, corneal hydration and ambient humidity factors could affect the final outcome with the conventional excimer laser systems, whereas they have a limited effect when the solid state laser is used [32, 33].

In the present study, a fourth harmonic wavelength of solid-state Nd:YAG pulsed laser is generated in order to analyze its efficacy for corneal reshaping procedure. The ablation effect of laser radiation at 266 nm has been studied on both ex-vivo rabbit cornea and PMMA as an equivalent corneal ablation material. In addition, histopathological effect on corneal stroma has been examined.

## Materials and methods

### Experimental setup

Continuum Minilite PIV Q-switched Laser source at 532 nm (second harmonic generation of 1064 nm Nd:YAG) with pulse width 4±1 ns, pulse energy 14 mJ and spot diameter 3 mm has been directed to a BBO nonlinear crystal (Fuzhou Hundreds Optics Inc, China) to generate fourth harmonic Nd:YAG laser radiation at 266 nm. The crystal dimensions are 4×4×10 mm^3^ with θ of 47.7° and φ of 0°, where θ is the angle between the incident wave vector and optic axis of the crystal and φ is angle between projection of incident wave vector on the x-y plane and the x axis of the crystal. The sides are coated with anti-reflection coating for 532 nm and 266 nm.

In order to separate the laser radiation at 266 nm from the emitted radiation at other wavelengths, UVFS pl mirror (EKSMA optics, UAB) has been utilized. This mirror diameter and thickness are 25.4 mm and 6 mm, respectively. The mirror shows a reflection efficiency of > 99.5% at 266 nm whereas the transmission efficiency is 99% at 532 and 1064 nm at an angle of incidence of 45°. The partial reflection of the 532 and 1064 nm beams has been eliminated using a 3.5-mm pinhole. A fused silica focusing lens of a focal length of 300 mm is used to collimate the laser beam to 0.7×0.4 mm^2^ (oval shape) in order to intensify the laser pulse for a significant photo-ablation effect.

**Fig 1:**
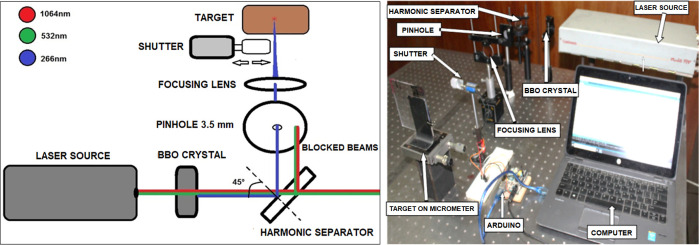
(a) Schematic diagram and (b) lab photo of the implemented setup.

For accurate count of number of pulses, a solenoid shutter is placed between the focusing lens and the target. The duration in which the shutter is open/closed is controlled using ARDUINO electronics platform. Finally, the target is placed on a micrometer XY translation stage for fine adjustment of the laser pulses locations on the PMMA [34]. Illustration and image of the experimental setup are presented in Fig 1(A) and 1(B), respectively.

Different numbers of pulses have been applied on both PMMA target and Rabbit cornea as listed in Table 1.

**Table 1:** Test conditions parameters.

	Pulse duration	Spot size	Pulse energy	Number of pulses
**PMMA**	3–5 ns	0.7×0.4 mm^2^	2.5 mJ	50, 100, 200, 300, 400, 500
**Rabbit eye**				50, 100

It should be noted that only number of pulses of 50 and 100 are histopathologically examined since higher numbers of pulses are not common in corneal reshaping procedure. The dimensions of the resultant ablation spots on the PMMA were determined using a polarized microscope (OLYMPUS, BX53). However, the shape of the resultant ablation on the rabbit cornea surface was imaged using a triocular zoom stereomicroscope (EXTRA+OPTECH, Germany) connected with DEC-2 digital camera.

### Ex-vivo sample collection, preparation, and placement

The present study does not involve any contact with alive animals. The samples including the rabbit eyes have been purchased from a local butcher shop in the vicinity of Cairo University campus (El-Orouba Birds, Zagazig St, Al Omraneyah Al Gharbeyah, El Omraniya, Giza Governorate) and transported to our laboratory at Cairo University in clean containers within 30 minutes after slaughtering for ex-vivo investigations. It should be noted that, cooked rabbit is an Egyptian dish and, hence, the rabbit eye samples do not require any specific ethical measures since they were already slaughtered for commercial food production. The rabbit eye is considered as animal disposals or remains.

**Fig 2:**
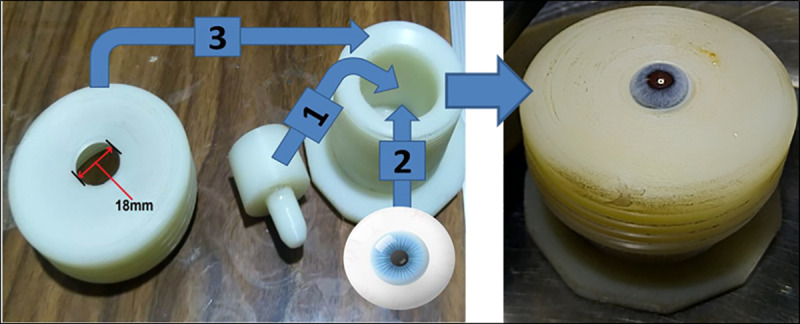
The designated globe holder.

To examine the ablation capability of the produced UV wavelength (266 nm) one the stroma, mechanical epithelium debridement of 7.5 mm at the central region of the cornea (previously marked with a trephine) was performed. A specific holder is designed to hold the eye globe during the removal of the epithelium layer and laser ablation process as well. This holder is made of Teflon locally in our lab and it consists of three parts assembled together with an opening of about 18 mm as illustrated in the Fig 2.

For investigating the histopathologic changes of rabbit’s cornea after the ablation process, eye samples were flushed and fixed in 10% neutral buffered formalin (NBF) for 72 hrs. Samples were then trimmed and processed in serial grades of ethanol and cleared in Xylene. Furthermore, eye samples were infiltrated and embedded into Paraplast tissue embedding media. A 4-μm thick sections were sliced by rotatory microtome and mounted on glass slides. Tissue sections were stained by Hematoxylin and Eosin as a general morphological examination staining method then examined via light microscope (Lecia Microsystems GmbH, Germany). All standard procedures for samples fixation and staining were performed according to Culling [35]. During the ex-vivo experiments, the humidity was 50% and temperature was 25°C and measured by digital temperature humidity meter (UT333S hygrometer, UNI-T, Poland).

## Results and discussion

### Ablation in PMMA

**Fig 3:**
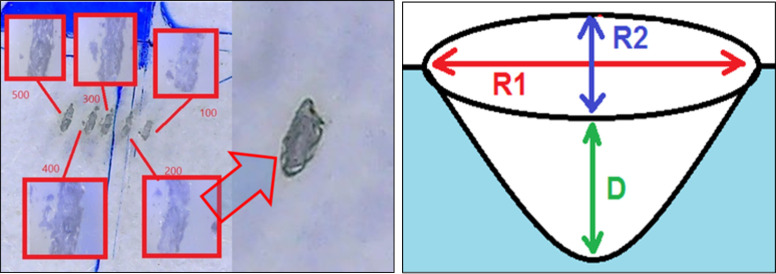
Resultant ablation on PMMA. (a) at different number of pulses, and (b) illustration of the cone-like shape in the depth direction.

Different numbers of laser pulses at 266 nm of a pulse energy of 2.5 mJ and spot size of 0.7 mm have been applied on the PMMA target resulting in an ellipse-like ablation shape as presented in Fig 3(A). The ablation depth increases with increasing the number of pulses resulting in a conical shape in the depth as shown in Fig 3(B).

**Fig 4:**
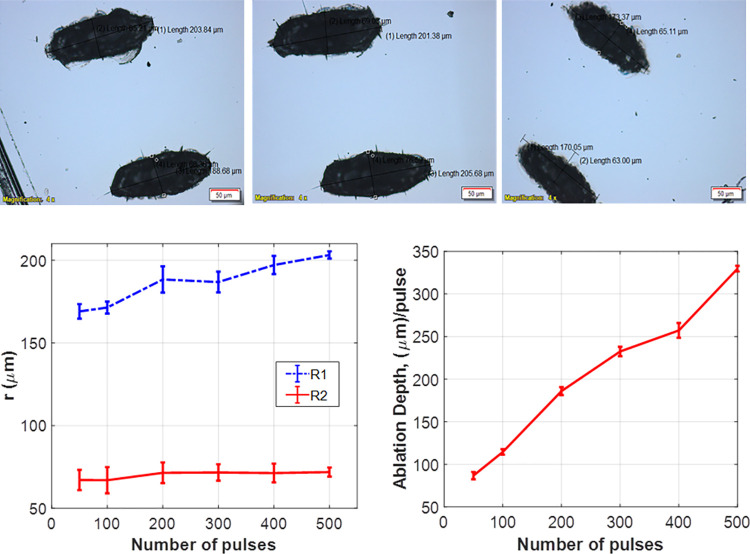
(a) Ablation in PMMA at different number of pulses obtained with polarized microscope, (b) variation of the ellipse diameters with the number of applied pulses, and (c) change in the ablation depth with the number of applied pulses.

The major and minor axes (R1 and R2, respectively) of the resultant ellipse-shaped ablation have been determined using the polarized microscope. Fig 4(A) presents samples of the obtained images. Each number of pulses has been repeated 6 times on different locations on the PMMA target and the variation in the major and minor axes has been plotted versus the applied number of pulses as illustrated in Fig 4(B). The ablation depth (D) is measured using binocular inverted microscope based on the refining displacement of its micrometer upon imaging the ablated spots on the PMMA. The change in the ablation depth with number of applied laser pulses is presented in Fig 4(C).

Ablation on the PMMA (Fig 4) showed that the minor axis R2 almost does not change with increasing the number of the pulses whereas the major axis R1 exhibits a slight increase. This reveals the stability of the emitted pulse energy over the studied range of pulse numbers as well as the homogeneity of the resultant beam profile. Moreover, the ablation depth D increases significantly with increasing the number of pulses which is a sign of proper ablation effect.

**Fig 5:**
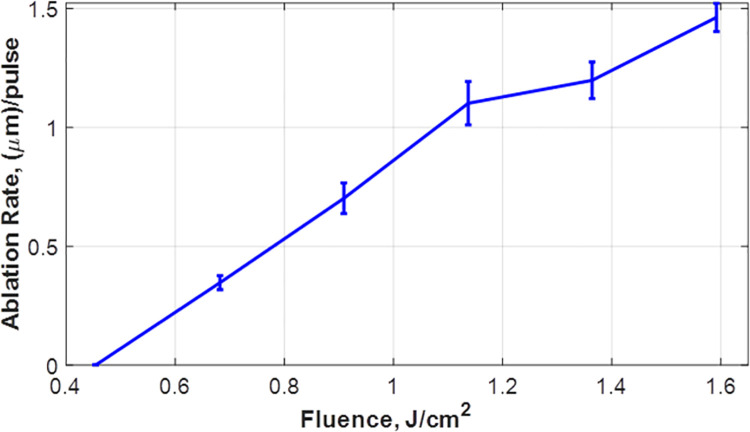
Ablation rate in PMMA at different fluence values.

The relation between ablation rate and fluence provided in Fig 5 reveals that the ablation rate almost linearly increases with the applied fluence which agrees with previous studies on the other UV wavelengths [27, 36–38]. Furthermore, the results show that the obtained ablation rate is comparable to that reported with excimer laser at 193 nm.

In order to determine the ablation threshold of PMMA, we reduced the pulse energy while keeping the number of pulses at 100 in each trial. The ablation rate (ablated depth in μm divided by the number of pulses) versus the fluence is presented in Fig 5.

**Fig 6:**
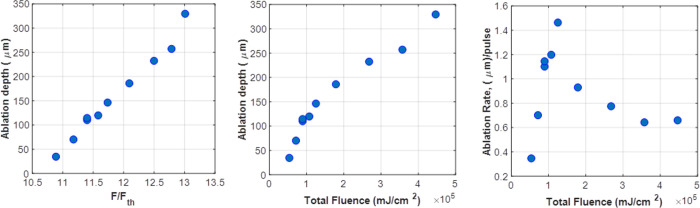
Total ablation depth as a function of (a) normalized fluence and (b) total fluence, and (c) variation in the ablation rate with the fluence.

On the first set of experiments, a constant energy per pulse (2.5 mJ) with a various number of pulses (from 50 to 500 pulses) was applied as previously presented in Fig 4. The use of such high fluence resulted in a reduced ablation rate as shown in Fig 6(C). The variation in the ablation depth with the normalized fluence and the total fluence is illustrated in Fig 6(A) and 6(B), respectively.

In the present study, a fluence of 1.1 J/cm^2^ has been used, which is higher than the previous studies. Nonetheless, it was choose to ensure proper ablation effect in PMMA and ex-vivo rabbit cornea since the absorption coefficient in PMMA and rabbit cornea is relatively lower at 266 nm than that at 193 nm. Despite the fact that the absorption coefficient at 266 nm is lower than 193 nm, the obtained ablation threshold using PMMA was approximately 328 mJ/cm^2^ and the obtained ablation rate was comparable to that obtained by other UV wavelengths [39]. The threshold fluence was 328 mJ/cm^2^ for the 100-pulses experiment which is slightly higher than that at 249 nm and lower than that at 308 nm as reported by Krueger et al. [40]. One possible reason for this observation is that the decomposed transient debris could have absorbed photons in 266 nm which will reduce the number of photons reaching the target.

It should be noted that the variation of threshold with laser repetition rate (in Hz) has not been investigated in the present study, however, the dynamic absorption coefficient (*α*) can be calculated following the blowup model from Beer-Lambert law as:

$$\alpha =\frac{1}{D}ln(\frac{F}{F_{th}})$$

where D is the ablation depth in cm, F_th_ is the ablation threshold, and F is the fluence. Based on the calculations, *α* value will be 7958.8 ± 0.014 cm^-1^. The computed dynamic absorption coefficient is not greater than that previously reported at 193 nm.

### Ex-vivo rabbit corneal ablation

**Fig 7:**
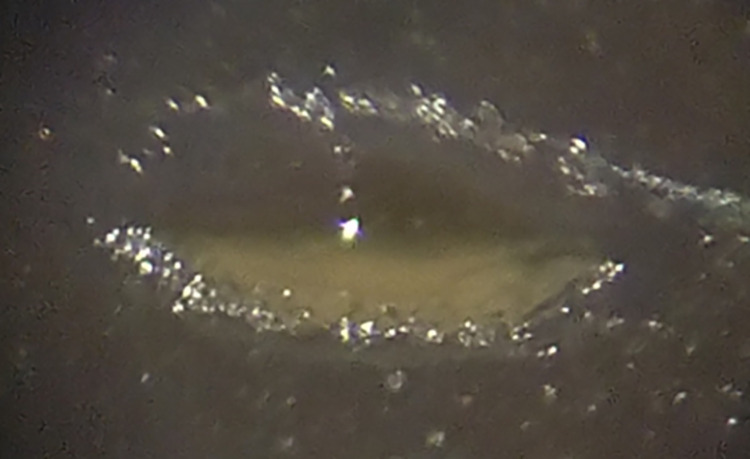
The resultant ablation in corneal tissue.

The ablation area in rabbit’s cornea has been screened with the triocular zoom stereomicroscope. The obtained results are presented in Fig 7.

**Fig 8:**
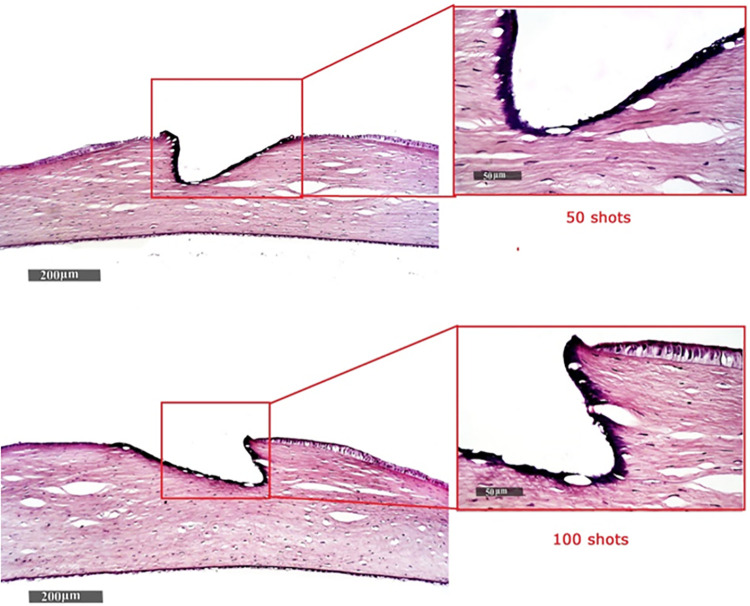
Histopathologic changes of corneal tissue after applying laser pulses. (a) 50 pulses, and (b) 100 pulses.

The obtained results of the histopathologic investigation (performed using light microscope) are presented in Fig 8.

Referring to ex-vivo rabbit cornea obtained ablation results, the incision is shown to be a clean-cut which indicates a relatively small thermal effect than that in the case of PMMA. Furthermore, the zone of thermal interaction is limited in the incision wall with no collateral damage as shown in Fig 7. Fig 8 shows minimal thermal damage and relatively smooth ablation surfaces at the targeted regions sides with no observed signs of edema or distortion of the adjacent corneal stroma. Activated keratocytes were observed in the stromal layers adjacent to the epithelium. However, the structure of the descemet membrane or the endothelial layer has not been affected. Consequently, the generated fourth harmonic Nd:YAG laser radiation (at 266 nm) showed a smooth ablation surface with no edema or distortion of the adjacent corneal stroma, indicating minimal thermal damage after PRK in rabbit eyes as compared with previous studies using the same wavelength [41, 42].

## Conclusions

Fourth harmonic of Nd:YAG laser has been generated using two nonlinear optical elements. The laser beam is applied on PMMA and Ex-vivo rabbit cornea with different number of pulses to investigate its photo-ablation effect. The PMMA showed elliptical ablated area with almost constant major and minor axes, while the depth (which has conical-like shape) significantly increases with increasing the number of pulses indicating stable pulse energy, homogenous beam profile, and proper ablation effect. The Ex-vivo rabbit stroma showed significant tissue undulation, minimal thermal damage, and relatively smooth ablation surfaces at the targeted regions. Consequently, the proposed results present a promising alternative to the 193-nm excimer laser for corneal ablation process. Replacing the ArF excimer laser (193 nm) with a comparable solid state laser is supposed to overcome the disadvantages of the traditional gas laser in addition to reducing its size. For future work, the histopathologic changes of rabbit’s cornea at different laser powers of the 266-nm laser radiation should be investigated. Additionally, an optimization of the parameters is needed in order to obtain the best ablation results with the minimum thermal effect.
